# Resolving the SLOSS dilemma for biodiversity conservation: a research agenda

**DOI:** 10.1111/brv.12792

**Published:** 2021-08-28

**Authors:** Lenore Fahrig, James I. Watling, Carlos Alberto Arnillas, Víctor Arroyo‐Rodríguez, Theresa Jörger‐Hickfang, Jörg Müller, Henrique M. Pereira, Federico Riva, Verena Rösch, Sebastian Seibold, Teja Tscharntke, Felix May

**Affiliations:** ^1^ Geomatics and Landscape Ecology Laboratory, Department of Biology Carleton University 1125 Colonel By Drive Ottawa ON Canada; ^2^ John Carroll University 1 John Carroll Blvd. University Heights OH U.S.A.; ^3^ University of Toronto – Scarborough 1265 Military Trail Toronto ON Canada; ^4^ Instituto de Investigaciones en Ecosistemas y Sustentabilidad Universidad Nacional Autonoma de Mexico Antigua Carretera a Patzcuaro No. 8701, Ex‐Hacienda de San Jose de la Huerta 58190 Morelia Michoacan Mexico; ^5^ Escuela Nacional de Estudios Superiores Universidad Nacional Autonoma de Mexico Tablaje Catastral No. 6998, Carretera Merida‐Tetiz km 4.5, Municipio de Ucu 97357 Merida Yucatan Mexico; ^6^ German Centre for Integrative Biodiversity Research (Halle‐Jena‐Leipzig) Deutscher Platz 5e 04103 Leipzig Germany; ^7^ Institute of Biology Martin Luther University, Halle‐Wittenberg Am Kirchtor 1 06108 Halle (Saale) Germany; ^8^ University of Würzburg Sanderring 2 97070 Würzburg Germany; ^9^ Bavarian Forest National Park Freyunger Str. 2 94481 Grafenau Germany; ^10^ Ecosystem Analysis, Institute for Environmental Science University of Koblenz‐Landau Fortstraße 7 76829 Landau Germany; ^11^ Ecosystem Dynamics and Forest Management Research Group Technical University of Munich Hans‐Carl‐von‐Carlowitz‐Platz 2 85354 Freising Germany; ^12^ Berchtesgaden National Park Doktorberg 6 83471 Berchtesgaden Germany; ^13^ Agroecology University of Göttingen Wilhelmsplatz 1 37073 Göttingen Germany; ^14^ Freie Universität Berlin Kaiserswerther Str. 16‐18 14195 Berlin Germany

**Keywords:** dispersal, edge effect, extinction–colonization, geometric effect, habitat fragmentation, landscape scale, SLOSS cube hypothesis, metacommunity, spatial sampling effect, species aggregation

## Abstract

The legacy of the ‘SL > SS principle’, that a single or a few large habitat patches (SL) conserve more species than several small patches (SS), is evident in decisions to protect large patches while down‐weighting small ones. However, empirical support for this principle is lacking, and most studies find either no difference or the opposite pattern (SS > SL). To resolve this dilemma, we propose a research agenda by asking, ‘are there consistent, empirically demonstrated conditions leading to SL > SS?’ We first review and summarize ‘single large or several small’ (SLOSS) theory and predictions. We found that most predictions of SL > SS assume that between‐patch variation in extinction rate dominates the outcome of the extinction–colonization dynamic. This is predicted to occur when populations in separate patches are largely independent of each other due to low between‐patch movements, and when species differ in minimum patch size requirements, leading to strong nestedness in species composition along the patch size gradient. However, even when between‐patch variation in extinction rate dominates the outcome of the extinction–colonization dynamic, theory can predict SS > SL. This occurs if extinctions are caused by antagonistic species interactions or disturbances, leading to spreading‐of‐risk of landscape‐scale extinction across SS. SS > SL is also predicted when variation in colonization dominates the outcome of the extinction–colonization dynamic, due to higher immigration rates for SS than SL, and larger species pools in proximity to SS than SL. Theory that considers change in species composition among patches also predicts SS > SL because of higher beta diversity across SS than SL. This results mainly from greater environmental heterogeneity in SS due to greater variation in micro‐habitats within and across SS habitat patches (‘across‐habitat heterogeneity’), and/or more heterogeneous successional trajectories across SS than SL. Based on our review of the relevant theory, we develop the ‘SLOSS cube hypothesis’, where the combination of three variables – between‐patch movement, the role of spreading‐of‐risk in landscape‐scale population persistence, and across‐habitat heterogeneity – predict the SLOSS outcome. We use the SLOSS cube hypothesis and existing SLOSS empirical evidence, to predict SL > SS only when all of the following are true: low between‐patch movement, low importance of spreading‐of‐risk for landscape‐scale population persistence, and low across‐habitat heterogeneity. Testing this prediction will be challenging, as it will require many studies of species groups and regions where these conditions hold. Each such study would compare gamma diversity across multiple landscapes varying in number and sizes of patches. If the prediction is not generally supported across such tests, then the mechanisms leading to SL > SS are extremely rare in nature and the SL > SS principle should be abandoned.

## I. INTRODUCTION

Conservation decision‐making relies on a combination of local knowledge and general rules or principles (reviewed in Gagné *et al*., 2015; Arroyo‐Rodríguez *et al*., 2020). Some of the first such general principles were proposed by Diamond (1975) for the design of nature reserves. One of Diamond's principles, inspired by MacArthur & Wilson's (1963, 1967) theory of island biogeography, was that a single large reserve (SL) should hold more species than several small reserves (SS) of the same total area, the ‘SL > SS principle’ [see also May (1975) and Diamond (1976)]. The SL > SS principle became a standard in conservation planning worldwide following its reiteration in the IUCN's (1980) highly influential *World Conservation Strategy*.

However, not all ecologists accepted Diamond's rationale for the SL > SS principle. Simberloff & Abele (1976) pointed out that the theory of island biogeography is in fact agnostic on the SLOSS question, i.e. “should conservation efforts be aimed at preserving a ‘single large or several small’ habitat patches?” (see also Simberloff & Abele, 1982). Indeed, the number of species on several small patches compared to one or a few large patches will depend on the degree to which species composition varies among the small patches, i.e. beta diversity (Higgs & Usher, 1980; Rösch *et al*., 2015). That is, the SLOSS question cannot be answered by comparing species richness on individual patches of different sizes; it must be addressed by comparing total species richness among sets of patches with the same total area but different numbers and sizes of patches.

Ecologists immediately began testing the SL > SS principle by comparing the number of species found in sets of habitat patches with the same total area but either a few large (SL) or several small (SS) patches. Early reviews of these empirical studies showed a lack of support for the principle. In particular, Simberloff & Abele (1982, p. 48) found “… not a single case where one large site unequivocally excels several small ones, and many cases where several small sites clearly contain more species than one large one”, and Quinn & Harrison (1988, p. 132) found that “[i]n all cases where a consistent effect of subdivision is observed, the more subdivided collection of islands or isolates contains more species.” In their review, Quinn & Harrison (1988) introduced the now‐classical SLOSS comparison method in which cumulative species richness *versus* cumulative area is plotted for a single set of patches, ordered from smallest to largest patch and from largest to smallest patch. If the smallest‐to‐largest curve lies above the largest‐to‐smallest curve then SS > SL, while SL > SS if the largest‐to‐smallest curve is above the smallest‐to‐largest curve. Such studies over the past three decades have continued to find SS > SL in most cases (reviewed in Fahrig, 2020).

In contrast to the frequent empirical result that SS > SL, theoretical work related to the SLOSS question suggests a more complex picture. Several hypotheses predict either SL > SS or SS > SL, depending on traits of the organisms (e.g. behaviour, life history) or the landscapes (e.g. habitat heterogeneity, disturbances). These are summarized below and in Table 1 (see also Ovaskainen, 2002; Fahrig, 2020). We also note that these conditions can occur together and may interact, resulting in a plethora of possible scenarios. Given this diversity of predictions, most ecologists have concluded that the answer to the SLOSS question ‘depends’ (Kingsland, 2002). For example, Sarkar (2012, p. 401) states that there is “no non‐contextual answer to the SLOSS question”, and the *Wikipedia* entry for ‘SLOSS debate’ concludes that “[t]he general consensus of the SLOSS debate is that neither option fit[s] every situation and that they must all be evaluated on a case to case basis.” The SLOSS debate has therefore largely disappeared from the ecological literature: *Google Ngram Viewer* indicates that the proportional occurrence of the term ‘SLOSS debate’ peaked in 2006 and has declined steadily since (see online Supporting Information, Appendix S1; Michel *et al*., 2011). More recent research for conservation planning is instead increasingly based on principles of representativity and complementarity that usually lead to the recommendation of multiple areas for conservation (Sarkar, 2012).

**Table 1:** Summary of theory and predictions related to the SLOSS debate; i.e. whether several small patches (SS) contain more species than a single (or few) large patches (SL) of the same total area (SS > SL), or the opposite (SL > SS). Note that many of the predictions require extrapolation from single species to multiple species. Superscript numbers identify studies that contributed to SLOSS‐relevant theory or to part of the theory

Ecological pattern	Prediction	Potential mechanisms
**I. Predictions based on extinction–colonization dynamics**		
*Assumption A. Variation in extinction rate dominates the outcome of extinction–colonization dynamics*.		
Extinction rate per patch decreases with increasing patch size.	SL > SS	Demographic stochasticity decreases with patch size.^a^ Species have minimum patch size requirements.^a^ Negative edge effects accentuate both of the previous mechanisms because patch edge‐to‐area ratio decreases with patch size. This disproportionately reduces patch size and increases demographic stochasticity for small patches compared to large patches.^b^ Higher per‐unit‐area emigration rate from small than large patches, due to higher edge‐to‐area ratio, leads to higher dispersal mortality in the matrix over SS than over SL.^c^
Extinction probability over the landscape is lower for SS than SL.	SS > SL	Between‐patch movements of a competitor/predator/parasitoid are lower than their within‐patch movements, and lower than between‐patch movements of the affected species. This results in spreading‐of‐risk to that species from antagonists, over SS.^d^ Disturbances cannot spread through the matrix, resulting in spreading‐of‐risk from disturbances over SS.^e^
*Assumption B. Variation in colonization rate dominates the outcome of extinction–colonization dynamics*.		
Colonization rates are higher across SS than SL.	SS > SL	Higher per‐unit‐area immigration rate over SS than SL due to: lower patch‐to‐patch distances in SS than SL; and higher edge‐to‐area ratio over SS than SL.^f^ Larger species pool available to SS than SL, due to the larger amount of habitat within an accessible distance of SS than SL.^g^
**II. Predictions based on beta diversity**		
Beta‐diversity is higher over SS than over SL.	SS > SL	Species distributions in continuous habitat are clumped due to: limited dispersal from occupied sites, conspecific attraction, and habitat heterogeneity. When patches are created by removal of habitat, SS intersect more pre‐existing micro‐habitats and species distributions than SL.^h^ Different successional trajectories in different patches produce higher heterogeneity and higher beta diversity over SS than SL.^i^

^a^ Skellam (1951); Diamond (1976); Whitcomb *et al*. (1976); Terborgh (1976); Cole (1981); Blake & Karr (1984); Willis (1984); Patterson & Atmar (1986); Burkey (1989); Atmar & Patterson (1993); Hill & Caswell (1999); With & King (1999); Etienne & Heesterbeek (2000); Pereira *et al*. (2004); McCarthy *et al*. (2006); Moilanen & Wintle (2007); Jagers & Harding (2009); Pardini *et al*. (2010); Tjørve (2010).

^b^ Preston (1960); Laurance (1991); Williams *et al*. (2005); Moilanen & Wintle (2007).

^c^ Willis (1984); Atmar & Patterson (1993); Fahrig (1998, 2002); Flather & Bevers (2002); Martin & Fahrig (2016).

^d^ Huffaker (1958); Levins (1969); Levins & Culver (1971); Simberloff & Abele (1976); Wiens (1976); Morrison & Barbosa (1987); Amarasekare & Nisbet (2001); Hernández‐Ruedas *et al*. (2018); Ben‐Hur & Kadmon (2020); Deane *et al*. (2020).

^e^ den Boer (1968); Levins (1969); Andrewartha (1984); Kallimanis *et al*. (2005); Tscharntke *et al*. (2008).

^f^ Dunning *et al*. (1992); Duelli (1997); Bowman *et al*. (2002*a*); Grez *et al*. (2004); Tischendorf *et al*. (2005); Puckett & Eggleston (2016); Fovargue *et al*. (2018); Fahrig *et al*. (2011).

^g^ Preston (1962); Tscharntke *et al*. (2012); Fahrig (2013).

^h^ Hutchinson (1959); Preston (1960); Diamond (1975); Higgs & Usher (1980); Margules *et al*. (1982); Nekola & White (1999); Kallimanis *et al*. (2005); Tjørve (2010); Socolar *et al*. (2016); May *et al*. (2019); Simberloff & Gotelli (1984); Lasky & Keitt (2013); del Castillo (2015); Socolar *et al*. (2016); Nekola & White (2002); Arroyo‐Rodríguez *et al*. (2017).

^i^ Laurance (2002); Laurance *et al*. (2007); Ewers *et al*. (2013); del Castillo (2015); Arroyo‐Rodríguez *et al*. (2017).

Despite the fact that most researchers have shelved the SLOSS debate, its legacy remains in practice, because many conservation agencies continue to prioritize protection of large, contiguous areas of habitat, while small patches of natural habitat are less likely to be protected (reviewed in Armsworth *et al*., 2018). For example, the current emphasis on ‘rewilding’ in Europe aims to conserve and restore large contiguous areas of natural habitat with at least a 10000 ha ‘core area’ (Europarc Federation, 2013). Three studies in Peru prioritized larger patches over smaller ones (Mindreau *et al*., 2013). Wetland conservation generally focuses on large wetlands, while most small wetlands around the world have little or no protection (reviewed by Hill *et al*., 2018). The same is true for small forest patches; forestry policy in Ontario, Canada, recommends cutting patterns that “defragment” the remaining forest by removing small patches (OMNR, 2001). And in Mexico, the Payment for Ecosystem Services program that offers payments to landowners to preserve their forest patches has recently increased the minimum patch area for eligibility from 25 ha (Hernández‐Ruedas *et al*., 2014) to 100 ha (CONAFOR, 2021), thus excluding most remaining forest patches from protection. In fact, Edwards, Fisher & Wilcove (2012) recommend preferentially clearing forest patches that are smaller than 1000 ha to meet future agricultural demand in the tropics, basing this recommendation on assumed low biodiversity value of small patches. The continued prioritization of large, contiguous habitat areas is also present in proposed guidelines of the High Conservation Value Resource Network for “identification of HCVs [high conservation values] globally, for any type of ecosystem, and across all natural resource sectors and standards” (Brown *et al*., 2013, p. iii). HCV 2 specifies “large landscape‐level ecosystems” and “intact forest landscapes”, implying that small ecosystems or forest patches (even in large numbers) have low conservation value. The HCV definition of a large ecosystem is context dependent but a “widely used” minimum size is 50000 ha (Brown *et al*., 2013, p. 30).

There are ecological, cultural, and practical arguments for the protection of large areas in some situations. When the choice is between one large area and one smaller one, the large one should usually be protected because it will generally contain more species than the smaller one. In addition, some conservation objectives other than total species richness may point to the protection of a large, contiguous area over many small ones. For example, for some individual species (e.g. some megafauna; Pe'er *et al*., 2014), sufficient habitat for population persistence may be only available in regions containing extensive, contiguous habitat. Large areas also may be needed to maintain the full ranges of some large‐scale natural ecosystem processes such as fire, flood, or disease dynamics (Perino *et al*., 2019). In addition, the cultural ecosystem service provided by the wilderness experience can require large, contiguous natural areas, although small areas can also have a ‘wildness’ value (Perino *et al*., 2019). Finally, in many situations it may be cheaper and easier to acquire and manage a few large patches than many small ones of the same total area (Armsworth *et al*., 2018).

Nonetheless, the assumed low value of small patches for biodiversity conservation is problematic in regions where most remaining habitat occurs only in small patches. These are often human‐dominated ecoregions where most natural habitat has been lost to human uses and there are few protected areas (e.g. Taubert *et al*., 2018; Hannah *et al*., 2020). For example, small patches of habitat in and around urban areas often contain rare species and have high biodiversity value (Planchuelo, Kowarik & von der Lippe, 2020). In such regions, the down‐weighting of the relative conservation value of small habitat patches undermines habitat preservation where protection of biodiversity is most needed (e.g. Ribeiro *et al*., 2009).

In summary, even though most ecologists have moved on from the SLOSS debate, the favouring of larger over smaller habitat patches in conservation suggests a need to clarify when SL > SS. Theory predicts SL > SS under certain conditions (Table 1), but so far these are not well supported in empirical studies (reviewed in Fahrig, 2020). In particular, we need to know whether the SL > SS principle is consistently and predictably valid over a defined set of ecological conditions. If it is not, then the mechanisms leading to SS > SL counterbalance or outweigh those predicted to lead to SL > SS. This would, in turn, suggest that the SL > SS principle should be abandoned.

Here we propose a research agenda to resolve the SLOSS dilemma, addressing the question, ‘are there consistent, empirically demonstrated conditions in which few large patches hold more species than several small ones?’ We begin by reviewing the relevant SLOSS theory and predictions (Table 1). We then propose a hypothesis, the ‘SLOSS cube hypothesis’, which summarizes existing SLOSS predictions and empirical work, and finally we use that hypothesis to propose a research agenda. Our aim is to encourage future research in a direction that will resolve the SLOSS dilemma.

## II. REVIEW OF SLOSS PREDICTIONS

The goal of our literature review was to find all theory, broadly defined, that has been used to make predictions about the SLOSS question. We began by searching on *Web of Science*, up to the end of 2019, using the following search string: (“several small” OR “several‐small” OR “SLOSS”) AND (“single large” OR “single‐large” OR “SLOSS”), refined by research area to environmental sciences and biodiversity and conservation. We retained all papers presenting SLOSS predictions, whether based on formal theory or verbal arguments. Although SLOSS is specifically about species richness, most of the predictions related to SLOSS are based on single‐species models and mechanisms (Ovaskainen, 2002), which are then extrapolated to species richness. Therefore, we retained both single‐species and multi‐species SLOSS theory. We also reviewed publications cited in the papers identified through our *Web of Science* search. We summarize the SLOSS predictions below and in Table 1.

### (1) Extinction–colonization‐based predictions where variation in extinction dominates

Most predictions of SL > SS derive from the assumption that variation in extinction rate dominates the outcome of the extinction–colonization dynamic. This is expected when populations in separate patches are largely independent of each other because movements among patches are rare such that colonization events are infrequent. Such isolation among patches should occur when: (*i*) patches are far apart; (*ii*) the matrix is hostile and leads to very high dispersal mortality; (*iii*) the species avoid entering the matrix (as might occur for habitat interior specialists); or (*iv*) the species have very low innate mobility. As small patches are expected to have smaller populations than large patches, they should have higher extinction rates from demographic stochasticity. The persistence of any given species on a set of isolated patches will then be driven by the size of the largest patch (Burkey, 1989; Etienne & Heesterbeek, 2000; Jagers & Harding, 2009). This effect will be accentuated for habitat interior species because the proportion of a patch that is interior habitat declines with decreasing patch size (Preston, 1960; Laurance, 1991; Williams, ReVelle & Levin, 2005; Moilanen & Wintle, 2007). This effect is also expected to be accentuated in situations where the matrix is hostile, for species that readily emigrate from patches. Emigration rate should be higher from SS than SL due to the larger edge‐to‐area ratio for SS, leading to lower retention of dispersers within natal patches in SS than SL. Therefore the mortality rate in the hostile matrix will be higher for SS than SL (Willis, 1984; Atmar & Patterson, 1993; Fahrig, 1998, 2002; Flather & Bevers, 2002; Martin & Fahrig, 2016). The SL > SS pattern is also predicted to be stronger when species within a group have different patch size requirements and small patches are smaller than the patch size requirements of some species, leading to selective extinction of particular species from small patches (Diamond, 1976; Terborgh, 1976; Cole, 1981; Patterson & Atmar, 1986; Atmar & Patterson, 1993; McCarthy, Thompson & Williams, 2006; Tjørve, 2010).

On the other hand, theory can predict SS > SL in extinction‐dominated systems where dispersal among patches is limited, when extinctions are caused by an antagonistic species or by a disturbance. This leads to spreading‐of‐risk of landscape‐scale extinction across SS. Division of habitat into many small patches is predicted to reduce interspecific competition, such that poorer competitors can persist on some small, isolated patches due to the absence of stronger competitors (e.g. Heilmann‐Clausen & Christensen, 2004; Hernández‐Ruedas *et al*., 2018). This could increase the overall number of species across a set of small patches, leading to SS > SL (Levins & Culver, 1971). SS can also stabilize predator–prey or host–parasitoid interactions as prey or hosts can escape to patches that are unoccupied by the predator or parasitoid (Huffaker, 1958; Levins, 1969; Wiens, 1976; Morrison & Barbosa, 1987). This should lead to SS > SL for groups of predators and their prey, or parasitoids and their hosts. In addition, SS are predicted to reduce the risk of simultaneous extinction due to disturbances that do not spread from patch to patch through the matrix (den Boer, 1968; Levins, 1969; Andrewartha, 1984; Kallimanis *et al*., 2005; Tscharntke *et al*., 2008), again leading to SS > SL.

### (2) Extinction–colonization‐based predictions where variation in colonization dominates

When movements among patches are common, population processes are generally predicted to lead to SS > SL (Table 1). SS should have a higher rate of colonization than SL, for two reasons: a higher immigration rate in SS than SL, and a larger species pool in the proximity of SS than SL. If a species has a very high rate of emigration from patches (e.g. larval fish; Fovargue, Bode & Armsworth, 2018) then, for most dispersal and habitat‐searching behaviours, SS are predicted to intercept more dispersers than SL because of the higher edge‐to‐area ratio of SS than SL (Bowman, Cappuccino & Fahrig, 2002*a*). This will lead to higher immigration and therefore higher colonization rates in SS than SL (Grez *et al*., 2004; Tischendorf *et al*., 2005; Puckett & Eggleston, 2016). In other words, in this situation SS are usually predicted to have higher functional connectivity (*sensu* Taylor *et al*., 1993) than SL. In addition, some species groups need to access specific resources that are not available in their breeding habitat patches, during some other part of their life cycle (e.g. amphibians that need wetlands during breeding and then move to upland habitats for feeding; Pope, Fahrig & Merriam, 2000). This could lead to SS > SL for the breeding habitat, because access to those other resources will generally be higher in a landscape where breeding habitat is distributed in many small patches [‘landscape complementation’ (Dunning, Danielson & Pulliam, 1992; Duelli, 1997; Fahrig *et al*., 2011)].

**Fig 1:**
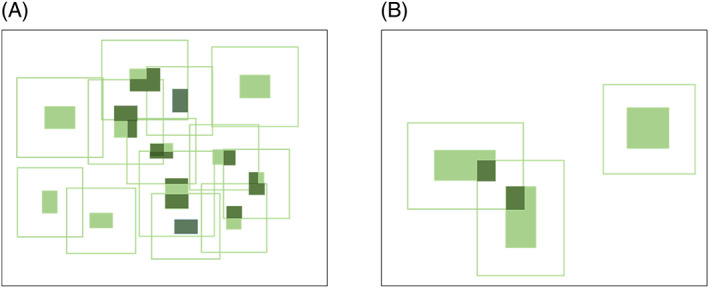
The total area contributing the species pool available to colonize a set of several small patches (A) is larger than the total area contributing the species pool available to colonize a set of few large patches of the same total area (B). Light‐coloured rectangles are patches. Boxes around them represent the areas from within which habitat can contribute colonists to the patches. Dark‐coloured rectangles are the areas of other habitat patches within the local landscape surrounding each patch.

The species pool available to colonize SS is also expected to be larger than the species pool available to colonize SL. If potential colonists can arrive at a patch from nearby habitat of the same type within a given distance of the patch (the patch's ‘local landscape’), then the species pool available to colonize a set of habitat patches (SS or SL) will depend on the total area of that same habitat type within their local landscapes. This total area of potential donor habitat is expected to be larger for SS than SL because the total edge length of SS is larger than the total edge length of SL, making the sum of the local landscapes larger for SS than for SL (Fig. 1). Along with the larger total habitat amount in the local landscapes of SS than SL will likely come more micro‐habitats within that habitat, i.e. higher habitat heterogeneity, further increasing the pool of potential colonizing species for SS compared to SL (Tscharntke *et al*., 2012).

### (3) Predictions based on beta diversity

All of the SLOSS predictions discussed so far derive from assumptions about how extinction/mortality and colonization/immigration dynamics interact with sizes of individual patches and with sets of patches. In general, SL > SS is predicted when extinction dominates these dynamics, except when spreading‐of‐risk plays an important role in population dynamics. SS > SL is generally predicted when colonization/immigration dominates the dynamics. Considering these factors together, Ovaskainen (2002) predicted that an intermediate number of medium‐sized patches will often hold the most species.

**Fig 2:**
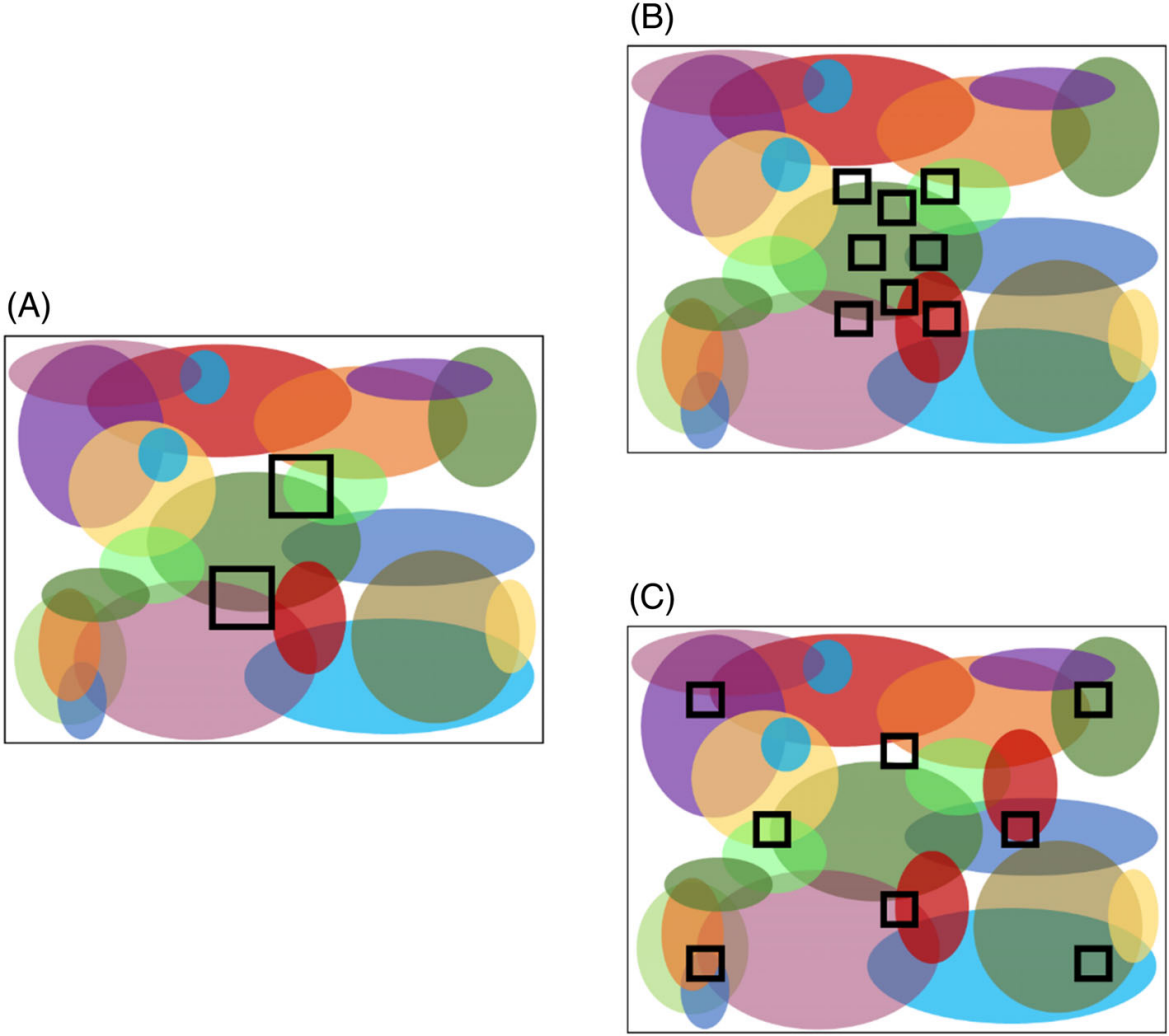
When species distributions are clumped or spatially autocorrelated, a few large patches (A) will intersect (sample) fewer species than several small patches (B and C). Different colours represent different species within continuous habitat in a single ecoregion before habitat loss (large rectangles). Squares represent patches subsequently created by habitat loss. When the landscape extent (maximum distance between patch edges) is the same for few large and several small patches (A *versus* B), several small patches will cover the area more evenly and will therefore intersect more species: in A two large patches intersect three species while in B eight small patches intersect five species. This effect is accentuated if the several small patches are further apart than the few large patches: in C eight small patches intersect nine species compared to three species in A.

A different set of SLOSS theory asks how the number of patches (for a given total habitat amount) is expected to affect beta diversity. Perhaps the simplest or null theory is the ‘geometric effect’, based on a cookie‐cutter analogy (May *et al*., 2019). Here, the species occurring in a habitat patch are simply those that existed there before it became cut into a patch through habitat loss. When these pre‐existing species distributions are clumped or spatially autocorrelated, a given area cut into a large number of small patches will intersect more of these pre‐existing species distributions than when that area is cut into a small number of large patches, leading to higher beta diversity in SS and a prediction of SS > SL (May *et al*., 2019; Fig. 2).

We consider the geometric effect a null expectation because most species distributions are clumped (Nekola & White, 1999; Tuomisto, Ruokolainen & Yli‐Halla, 2003; Seidler & Plotkin, 2006; Morlon *et al*., 2008; McGill, 2010, 2011). Clumping can arise from intrinsic factors including limited dispersal from occupied sites creating population centres (Hubbell, 2001; Tuomisto *et al*., 2003), conspecific attraction (e.g. Vité & Francke, 1976; Ramsay, Otter & Ratcliffe, 1999; Schuck‐Paim & Alonso, 2001; Peignier *et al*., 2019), and philopatry (Weatherhead & Forbes, 1994). However, the main driver of clumped species distributions may be their responses to clumped or spatially autocorrelated micro‐habitats (e.g. soil type or microclimate). The ‘cookie‐cutter’ argument (above) also applies to micro‐habitats, and so we expect that a given area cut into a large number of small patches will intersect more micro‐habitats (higher across‐habitat heterogeneity) than when that area is cut into a small number of large patches. If species distributions are related to micro‐habitat distributions, this leads to a prediction of SS > SL due to higher beta diversity in SS (Lasky & Keitt, 2013). An exception to this argument would occur if large patches contain specific micro‐habitats that are absent from all small patches, as might occur for island systems where maximum elevation is higher on large than on small islands. Finally, spatially autocorrelated disturbances are predicted to increase the spatial clumping or autocorrelation of micro‐habitats and species distributions, accentuating beta diversity in SS and the tendency for SS > SL (Diamond, 1975; Simberloff & Gotelli, 1984; Nekola & White, 2002; Kallimanis *et al*., 2005; Laurance *et al*., 2007; Lasky & Keitt, 2013).

Note that the prediction of higher beta diversity across SS than SL does not assume or imply that small patches are further apart than large patches. Indeed, when the landscape size and the amount of habitat are held constant, there is no consistent difference between SS and SL in habitat spread, measured as maximum habitat edge‐to‐edge distance (Appendix S2). Spatial clumping of species distributions is nevertheless expected to lead to more species sampled over SS than SL because SS will cover a given area more evenly than SL, thus intersecting more micro‐habitats and more species distributions leading to higher beta diversity [compare Fig. 2A and B (Tscharntke *et al*., 2002; May *et al*., 2019)].

If a set of several small patches happen to be more spread out than a set of a few large patches (e.g. Hill *et al*., 2011), beta diversity is predicted to increase even more over SS, due to even higher across‐habitat heterogeneity in SS than SL (Nekola & White, 1999; Morlon *et al*., 2008; Anderson *et al*., 2011; compare Fig. 2A and C). In other words, as pointed out early on by Higgs & Usher (1980), we can expect SS > SL if the proportional species overlap between patches is lower for SS than SL. However, increasing the distance among SS is also predicted to reduce inter‐patch movements, increasing patch isolation, and therefore to increase extinction dominance of extinction–colonization dynamics (Table 1). Thus, the spatial spread of SS is predicted to have two opposing effects: (*i*) decreasing inter‐patch movement potentially leading to SL > SS, and (*ii*) increasing across‐habitat heterogeneity and decreasing species overlap leading to higher beta diversity and SS > SL (Tjørve, 2010; Blowes & Connolly, 2012; Arnillas *et al*., 2017). Finally, higher beta diversity across SS than across SL may be accentuated over time following patch creation if different patches follow different successional trajectories (Laurance, 2002; Laurance *et al*., 2007; Ewers *et al*., 2013; del Castillo, 2015; Arroyo‐Rodriguez *et al*., 2017).

## III. RESEARCH AGENDA

Here we propose a research agenda for resolving the SLOSS dilemma. We first develop a hypothesis, the ‘SLOSS cube hypothesis’, that summarizes SLOSS predictions in combination with empirical SLOSS studies. We then use this hypothesis to propose an agenda for future empirical studies to ask, ‘are there any consistent, empirically demonstrated conditions that lead to SL > SS?’

### (1) The SLOSS cube hypothesis

**Fig 3:**
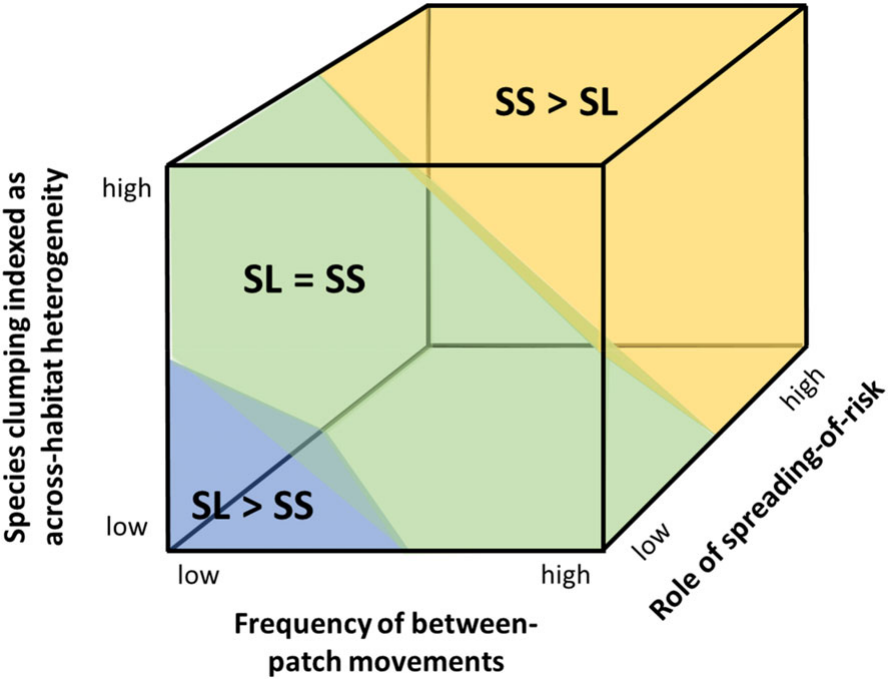
Illustration of the ‘SLOSS cube’, combining SLOSS‐relevant theory and empirical SLOSS studies. The axes are based on the theory and predictions summarized in Table 1. The proportional volumes of the three outcomes are based on their proportions found in a review of empirical SLOSS studies in which sampling effort was unbiased, i.e. sampling proportional to area (see fig. 2b in Fahrig, 2020): 50% SS > SL (yellow); 40% SL = SS (green); 10% SL > SS (blue). The SLOSS cube hypothesis predicts that SL > SS will dominate only when *all* of the following are true: between‐patch movement rate is low, the influence of spreading‐of‐risk on population dynamics is low, and across‐habitat heterogeneity is low, leading to low beta diversity.

The major features of SLOSS predictions and data to date are illustrated in Fig. 3. To summarize the predictions: (*i*) arguments based on population processes generally predict SL > SS when between‐patch movements are assumed to be rare, such that variation in local (patch) extinctions dominates the extinction–colonization dynamic; (*ii*) an exception to this occurs where spreading‐of‐risk plays a large role in population persistence, leading to a prediction of SS > SL; (*iii*) arguments based on population processes generally predict SS > SL when between‐patch movements are assumed to be common such that variation in colonization dominates the extinction–colonization dynamic; and (*iv*) consideration of beta diversity and across‐habitat heterogeneity generally leads to predictions of SS > SL. Therefore, SLOSS predictions can be largely characterized by the combination of three variables: the frequency of between‐patch movements; the role of spreading‐of‐risk in landscape‐scale population persistence; and the level of species clumping indexed as across‐habitat heterogeneity. These are the three axes in Fig. 3.

To summarize empirical work to date based on classical SLOSS studies (Quinn & Harrison, 1988): about 50% of apparently unbiased (see Section III.2) empirical SLOSS studies find SS > SL and about 10% find SL > SS, while the remaining 40% find no difference (Fahrig, 2020). These proportions are represented as the coloured volumes in Fig. 3. Given the relative rarity of SL > SS results, the SLOSS cube hypothesis predicts that SL > SS will occur predictably only when *all* of the following are met: between‐patch movements are rare, the role of spreading‐of‐risk in landscape‐scale population dynamics is low, *and* across‐habitat heterogeneity and species clumping are low, reducing the role of beta diversity (blue volume in Fig. 3). The prediction that all three conditions must hold to obtain SL > SS derives not only from the relative rarity of SL > SS, but also from preliminary summaries of relevant empirical work suggesting that when only one or two of these conditions holds we still find a predominance of SS > SL (reviewed in Fahrig, 2020). In particular, SS > SL is more common than SL > SS in situations with higher matrix hostility, suggesting that low between‐patch movement rate alone is insufficient to produce SL > SS reliably. In addition, many cases of SS > SL occur in situations where across‐habitat heterogeneity is low (Fahrig, 2020), suggesting that low spatial autocorrelation in environmental characteristics (leading to low beta diversity) alone is not sufficient to produce SL > SS reliably. Furthermore, a recent microcosm experiment involving 11 species over multiple trophic levels found SS > SL, even though there was no movement at all between patches and all habitat was homogeneous (Hammill & Clements, 2020). Extinction rates were lower across SS than SL, leading to SS > SL. This finding suggests that the effect of spreading‐of‐risk may be stronger than is often assumed and may be sufficient to result in SS > SL even when between‐patch movement is very low and SS are not more heterogeneous than SL. Consistent with this we note that 16 of 20 SLOSS comparisons from island systems reviewed in Fahrig (2020) found SS > SL. In these systems, between‐patch movement is low and SL are likely *more* heterogeneous than SS (see Section II.3), again suggesting a strong role of spreading‐of‐risk in creating SS > SL. Nevertheless, the *combination* of factors – low between‐patch movement, low role of spreading‐of‐risk, and low across‐habitat heterogeneity – has not yet been explicitly tested across a range of systems.

### (2) Testing the SLOSS cube hypothesis

Testing the SLOSS cube hypothesis will require a large number of individual empirical studies, where each study represents a point within the cube in Fig. 3. For each study, four variables should be estimated: (*i*) the frequency of between‐patch movements, (*ii*) the degree to which spreading‐of‐risk is important for landscape‐scale population persistence, (*iii*) the level of across‐habitat heterogeneity; and (*iv*) gamma diversity over different landscapes characterized by SS *versus* SL. Note empirical tests will measure across‐habitat heterogeneity rather than spatial clumping of species because estimating the spatial distribution of multiple species over multiple landscapes (see Section II.3) is generally not feasible. By contrast, across‐habitat heterogeneity is one of the major reasons for clumped species distributions, and can be measured from continuous raster maps based on remotely sensed data, using surface metrics (Riva & Nielsen 2020) such as metrics of spatial variance of the normalized difference vegetation index (NDVI; e.g. Duro *et al*., 2014).

The frequency of between‐patch movements is unknown and hard to measure for nearly all species groups and regions. While relative innate mobility of different species groups can often be estimated using morphological or life‐history correlates (Bowman, Jaeger & Fahrig, 2002*b*; Stevens *et al*., 2014; Beckman, Bullock & Salguero‐Gόmez, 2018), the realized frequency of between‐patch movements is related not only to innate mobility but also to landscape attributes such as habitat configuration and matrix quality. Therefore, we propose a combination of two steps for selecting species groups with low (or high) between‐patch movement frequency in the selected region. First, species groups would be identified as low or high innate mobility based on morphology and life‐history traits such as wing presence/absence, territory size, etc. The purpose of the second step is then to confirm that, in the selected region, the low‐mobility group does in fact show low between‐patch movement and/or the high‐mobility group does in fact show high between‐patch movement. This can be done indirectly by comparing mean species density (number of species per sample site) of the mobility group in landscapes with SS *versus* SL. If, for the low mobility group, movement is lower between patches than within patches in the selected region, then we should find lower mean species density in sample sites across a landscape with SS than across a landscape with SL (Tjørve, 2010).

The role that spreading‐of‐risk plays in landscape‐scale population persistence is also unknown for most groups of species. However, it should be possible roughly to categorize species groups into those that are likely to benefit from spreading‐of‐risk *versus* those that are not. Those that might benefit from spreading‐of‐risk would include: groups under strong top‐down control from predators/parasitoids; groups of weaker competitors; and groups subject to frequent local disturbances (see Section II.1). These conditions do not guarantee that the species group benefits from spreading‐of‐risk. However, the absence of all three of these conditions would be a strong indicator of a species group that does not benefit from spreading‐of‐risk, and therefore of a potential species group to include in tests of the question, ‘are there any consistent, empirically demonstrated conditions that lead to SL > SS?’ as illustrated in Fig. 3.

**Fig 4:**
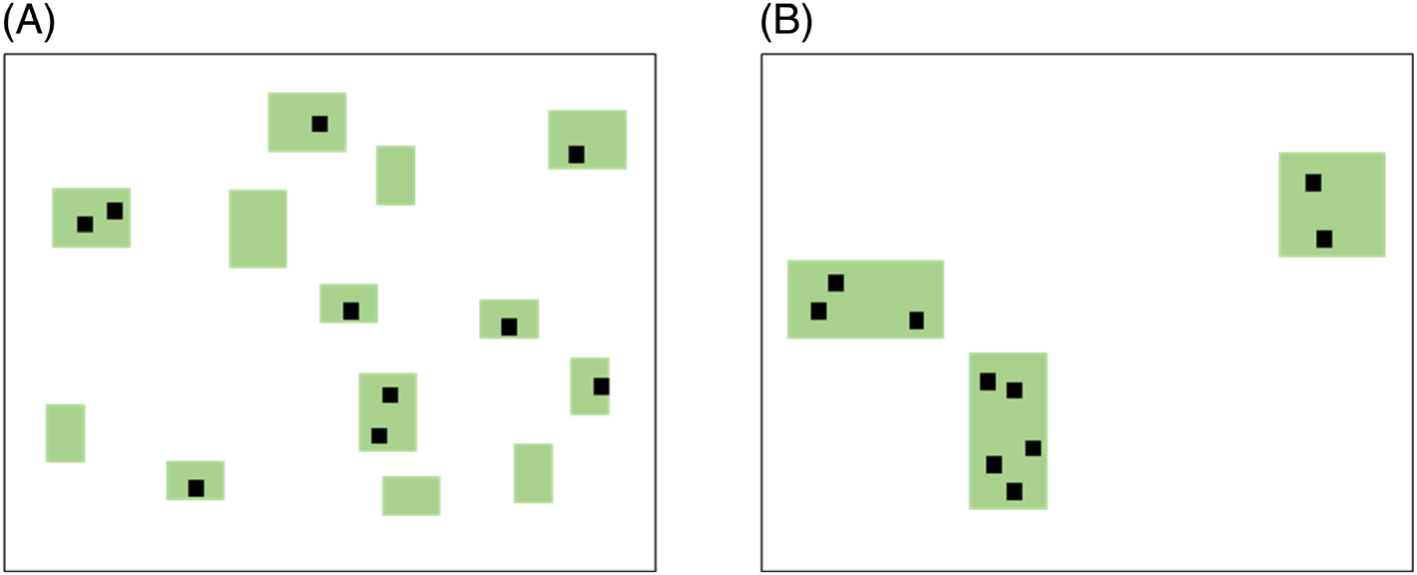
SLOSS can be evaluated by comparing cumulative species richness across the same number of sample sites (black squares) randomly placed within habitat (green rectangles) in multiple landscapes of the same size, each containing the same total area of habitat, but distributed in different numbers and sizes of patches. Two example landscapes are shown here, each with 10 sample sites placed randomly in habitat. Note that when the landscape has many small patches, some will not be sampled. This is not a problem because the unit of analysis in such a study is the landscape, not the patch.

Here we describe the characteristics of individual empirical studies that together would test the SLOSS cube hypothesis (Fig. 3). Importantly, each study should be based on randomly distributed samples within each of multiple landscapes (Fig. 4), rather than using the classical SLOSS approach (Quinn & Harrison, 1988) where species lists are combined across subsets of patches within the same landscape. Using random samples across multiple landscapes is preferable because it avoids two problems inherent in many empirical SLOSS studies to date.

First, using multiple landscapes avoids the problem that when all sampled patches are within the same landscape, large patches are intermixed with small patches. This classical study design is inconsistent with the inferences actually made from those studies, which are about whether many small patches (alone) have more or fewer species than few large patches (alone). In addition, when SLOSS is evaluated using subsets of intermixed patches, the link between the data and several of the mechanisms in Table 1 becomes unclear. For example, it is not clear how the spreading‐of‐risk of predation over SS would play out in a landscape in which small and large patches are intermixed. As another example, when small and large patches are intermixed, the amount of habitat contributing the species pools for colonization of SS *versus* SL (Fig. 2) includes portions of large and small patches within the local landscapes, and so the link between SLOSS and landscape moderation effects becomes unclear. Therefore, future empirical studies should sample species in multiple landscapes, each containing either SS or SL (Fig. 4), rather than subsets of SS or SL drawn from within a single landscape. The total habitat amount should be either the same across sampled landscapes, or at least habitat amount should be uncorrelated with the number of patches.

The second reason that using random samples across multiple landscapes is preferable to the classical SLOSS study design is that it avoids the problem that sampling is often biased in favour of SS (Gavish, Ziv & Rosenzweig, 2012). Small patches often have more sample sites per area than large patches, which means that the probability of detecting a given species is higher across SS than SL. Only about half of all SLOSS studies to date have apparently unbiased sampling effort (Fahrig, 2020). This is due to logistical constraints when the range of patch sizes is large. For example, if patches range in size from 5 ha to 1000 ha then, for equal sampling effort, the smallest possible number of sample sites in a 1000‐ha patch would be 200, assuming there is only one sample site per 5‐ha patch. For many species groups, such sample sizes would be impossible to accomplish. We note that if sampling effort information is available, patch size dependence on sampling effort can be estimated and controlled for in statistical models when using existing data (e.g. Deane *et al*., 2020). However, when designing a new empirical SLOSS study, the sampling effort problem can be best avoided by using random samples across multiple landscapes (Fig. 4). Cumulative number of species (gamma diversity) can then be directly compared for landscapes with SS *versus* landscapes with SL (Fig. 4).

Studies that together would test the SLOSS cube hypothesis should, ideally, have the following additional attributes. First, all sample landscapes in a given study (one point in the cube; Fig. 3) should have the same spatial extent and samples should be randomly distributed within the habitat in each landscape, with the number of samples in proportion to total habitat amount in the landscape. All sample landscapes within a study should also be within a single ecoregion, to ensure the same overall species pool, and the species included in the surveyed group should be those that are mainly associated with the particular habitat type studied. Here, significant attention should be paid to the definition of ‘habitat’. For example, single trees may not be habitat patches for species groups that rely on humid forest understorey conditions; however, single trees can be habitat patches for wood‐boring beetles. In addition, confounding of other variables with the SL *versus* SS comparison should be avoided. For example, an apparent pattern of SS > SL could be created where larger patches are more intensively managed or where smaller patches have more varied management approaches (e.g. grazed, mown, abandoned) than large ones, e.g. due to different ownership of different patches (Rösch *et al*., 2015). Conversely, an apparent pattern of SL > SS could be created where small patches are more disturbed by humans than are large patches (e.g. Barlow *et al*., 2016). In addition, the spatial pattern of patches should have been already in place for several generations of the surveyed species group. This is to ensure: (*i*) dissipation of transient positive fragmentation effects caused by a crowding effect on small patches following patch creation (Grez *et al*., 2004), and (*ii*) sufficient time for any extinctions to play out (Figueiredo *et al*., 2019). Finally, differences in species detectability (MacKenzie *et al*., 2002) between SS and SL should be estimated and accounted for if present. We reiterate that these are the ideal attributes for each study; where particular attributes cannot be controlled through study design, it may often be possible to control for them in statistical models.

### (3) Method for determining whether there are consistent, empirically demonstrated conditions that lead to SL > SS

As discussed above, the SL > SS principle continues to guide conservation decision‐making in many situations, despite lack of empirical support for it as a general principle. To resolve this dilemma, we need to determine whether SL > SS is in fact a valid principle in a predictable set of conditions. The principle could then be reworded as, ‘in general SL > SS whenever conditions *x* hold.’ Such conditions have been suggested (Table 1; summarized in Fig. 3) but to date there is little supporting empirical evidence. Therefore, resolving the SLOSS dilemma means addressing the question, ‘are there any consistent, empirically demonstrated conditions that lead to SL > SS?’

Addressing this question requires multiple empirical tests, using appropriate study designs, focused on species groups and environments where between‐patch movements are rare, spreading‐of‐risk is likely unimportant, and habitat is relatively homogeneous. In other words, studies should focus on the blue portion of the SLOSS cube in Fig. 3. If the majority of studies in this space find SL > SS, then we can conclude that the SL > SS principle is generally valid in those conditions. As discussed above, estimates of realized interpatch movement rates for groups of species are usually not available for a given region, but movement rates should be low when patches are very far apart or the matrix is hostile, or when the species group is comprised of sedentary species. Therefore, SLOSS tests should focus on regions where (*i*) habitat is rare, i.e. patches – both large and small – are far apart relative to the dispersal range of the species group, (*ii*) habitat is spatially homogeneous, and (*iii*) the matrix is hostile (e.g. urban areas, high‐intensity agriculture). The groups of species selected should be those assumed to have low innate mobility, and those for whom spreading‐of‐risk likely plays a minor role in population dynamics, i.e. stronger competitors under bottom‐up control that are not subject to frequent local disturbances (see Section II.1). Once a region and a species group have been selected, multiple sample landscapes should be selected within that region, that vary in the numbers and sizes of patches and do not vary in total habitat amount (Fig. 4), or for which there is no relationship between habitat amount and the numbers and sizes of patches across the landscapes. Sample sites should then be randomly placed in the habitat within each landscape, and the species group sampled at each site. Average species density (mean number of species per site) should be compared between SS and SL to confirm the assumption of low between‐patch movement in the selected region (see Section III.2). Total species richness (gamma diversity) should then be estimated across the habitat in each landscape to determine whether there are more species in landscapes with SL than SS.

### (4) Note on negative edge effects and SLOSS

As indicated in Table 1, habitat interior species, i.e. those that show negative edge effects, should be particularly susceptible to the effects of patch size on extinction probability. In addition, they may have low mobility between patches if they are averse to leaving interior habitat and entering the matrix. For this reason, patch‐scale evidence of negative edge effects on a species group is often taken as evidence of SL > SS for that group (Fletcher *et al*., 2018). However, we note that SL > SS cannot be directly inferred for groups of habitat interior species based only on patch‐scale evidence. Such an inference would entail cross‐scale extrapolation (Wiens, 1989) from local edge effects to landscape‐scale effects. This extrapolation is a prediction that must be tested at a landscape scale because other mechanisms in Table 1, operating at a landscape scale, may outweigh negative local edge effects in influencing species richness across a landscape (Fahrig *et al*., 2019). Such tests would compare gamma diversity of species groups known to show negative edge effects (and thus assumed to fall into the blue portion of the SLOSS cube in Fig. 3), across multiple landscapes as in Fig. 4.

We note further that such studies will need to estimate gamma diversity, not species richness at a sample site, i.e. species density. For example, Püttker *et al*. (2020) documented negative effects on species density of edge density in the local landscapes surrounding sample sites. The observed reductions in local richness cannot be directly extrapolated to infer SL > SS because other mechanisms, such as higher beta diversity across SS than SL, might outweigh the negative effect of edge density when species richness is measured over the landscape. Again, extrapolation to SL > SS needs to be tested by comparing species richness (gamma diversity) across multiple landscapes with different numbers and sizes of patches.

### (5) Need for many studies

Here we emphasize that a single study on a particular species group in a particular region cannot answer the question ‘are there consistent, empirically demonstrated conditions that lead to SL > SS?’ SL > SS was conceived and is used as a general principle. We know it is not universally valid, because most empirical studies do not support it. However, it may still apply in general when certain conditions hold, specifically the combination of low between‐patch movement, low spreading‐of‐risk, and low across‐habitat heterogeneity. Testing this prediction will require multiple studies on a range of taxa and regions that match these conditions (Seibold *et al*., 2018). SL > SS would be upheld as a principle if we find more species in habitat within landscapes with SL than in those with SS, in most of these studies.

## IV. DISCUSSION

In one sense, by proposing this research agenda, we are reviving a debate that most ecologists had set aside. Most ecologists believe that there is no general SLOSS principle and that each case must be evaluated individually. But at the same time the idea persists that the SL > SS principle is generally valid under some conditions (Table 1), such as for groups of habitat‐interior species and in landscapes with low matrix quality (Pfeifer *et al*., 2017; Fletcher *et al*., 2018), and that these are the conditions where biodiversity is most threatened. However, empirical tests to date do not generally support the SL > SS principle even in these conditions. If anything, the evidence so far suggests the reverse (Fahrig, 2020), although the number of tests is much smaller than needed for evaluating a general principle. We suggest that this contradiction needs to be resolved for the sake of biodiversity conservation, and we propose our research agenda as a path to resolving it.

We acknowledge, however, that testing the SLOSS cube hypotheses will be challenging, for three major reasons. First, each study requires sufficient information about the species in the study group, such as their primary habitat associations, their mobility, and their dominant interactions with other species. The second major challenge will be selecting multiple, appropriately sized landscapes to create cross‐landscape variation in the number of patches while minimizing potential confounding variables. Third, once the species group and landscapes have been selected, sampling many species across many sites within each of many landscapes will present a large logistical challenge. Nevertheless, we hope that the SLOSS cube hypothesis will allow researchers to identify study systems that are not only feasible but also will provide informative tests of the hypothesis. Recent improvements to habitat information through remote sensing developments (Skidmore *et al*., 2021), and large‐scale species sampling through community science (e.g. eBird, eButterfly) and through large, collaborative research efforts (e.g. Sirami *et al*., 2019) should increase the feasibility of tests going forward. Creative experimental approaches such as microcosm experiments (e.g. Hammill & Clements, 2020) may also be particularly useful for controlling the large number of factors involved. Overall, we recommend that particular effort should be put toward identifying and studying systems that are likely in the blue portion of Fig. 3, to focus tests on the question, ‘are there consistent, empirically demonstrated conditions leading to SL > SS?’

There are two possible outcomes of research aimed at testing the SLOSS cube hypothesis. First, empirical studies might generally support the hypothesis. This would have different implications for conservation decision‐making, depending on the conservation goal. In situations where the goal is general conservation of biodiversity, support for the hypothesis would suggest that a mixed strategy of mainly small patches and a few large patches would maximize biodiversity, as suggested by Arroyo‐Rodríguez *et al*. (2020). On the other hand, when the goal is the conservation of a particular species group, then support for the hypothesis would indicate what research the conservation agency needs to carry out to determine whether the particular conditions in their system fall within the range of conditions where SL > SS is valid. In particular, does the level of between‐patch movement, the role of spreading‐of‐risk, and the level of across‐habitat heterogeneity place the system within the blue portion of the SLOSS cube in Fig. 3? If so then, for biodiversity conservation, large patches should be prioritized and small patches should be down‐weighted, but if not, then total habitat amount and its heterogeneity should be maximized irrespective of the sizes of patches comprising it.

The second possible outcome of the proposed research agenda is that the majority of studies find either SS > SL or SL = SS throughout the SLOSS cube in Fig. 3, even when between‐patch movement, spreading‐of‐risk, and across‐habitat heterogeneity are all low. In that case, we should conclude that the SLOSS cube hypothesis is not supported, i.e. SL > SS is not a general principle under any predictable conditions, and therefore it should not be used in conservation planning. Two lines of evidence suggest this outcome is at least possible. First, reviews of empirical studies to date have not found predictable conditions leading to SL > SS; the majority of results so far find SS > SL for habitat specialists, when the matrix is hostile, when habitat amount is low, and when across‐habitat heterogeneity is low (reviewed in Fahrig, 2017*a*, 2020). Second, simulations by Fronhofer *et al*. (2012) suggest that the main mechanism proposed to lead to SL > SS – extinction–colonization dynamics dominated by variation in extinction rate – is likely very rare in nature. Fronhofer *et al*. (2012) predict this situation is usually evolutionarily unstable, with systems either becoming extinct or between‐patch movement rates increasing such that variation in extinction rate no longer drives the extinction–colonization dynamic.

We note that the idea that the SL > SS principle could be abandoned seems to fly in the face of the fact that there are documented empirical cases of SL > SS. Nevertheless, given the small number of these cases to date, if they do not occur in predictable conditions, then we would not be able to discount the possibility that they are due to statistical chance alone. In a review of effects of fragmentation *per se* (of which SLOSS is one component), Fahrig (2017*a*) found that 24% of significant fragmentation effects were negative (i.e. SL > SS in the context of SLOSS). However, fewer than 30% of all effects were significant, suggesting that fewer than 7.2% of all effects are significantly negative. Furthermore, documented reporting biases (Fahrig, 2017*b*) reduce this estimate to about 3–4.2% of all tests. Thus, if it turns out that there are no empirically demonstrated, consistent conditions leading to SL > SS, then it would be reasonable to infer that the few SL > SS findings to date may be due to statistical chance alone.

If there are no consistent conditions leading to SL > SS, this would confirm that small habitat patches have the same or greater biodiversity value as the same area of habitat in large patches. It would also mean that the overall goal for conservation should be to preserve or restore as much area as possible of each natural habitat type within a given ecoregion, intersecting the distributions of as many species as possible, irrespective of the patch sizes within which the habitat is distributed, as long as the patches are large enough to function as habitat for the species group (Rösch *et al*., 2015). It would also call into question conservation planning algorithms that minimize total boundary length, because their solutions favour larger, more compact areas, at the expense of the total area included in the ‘optimal’ solution (Stewart & Possingham, 2005; Hermosa *et al*., 2011). Removing the constraint that a given amount of habitat must be in large contiguous patches would increase options for conservation, especially in regions dominated by people where there are no large, contiguous natural areas remaining and where many taxa are declining (e.g. van Klink *et al*., 2020). Collections of small reserves such as small forest patches and riparian and wetland buffers could have high biodiversity value in such ecoregions. This would provide a rationale for local small‐scale conservation efforts, both public and private (Monteferri, 2019; Shumba *et al*., 2020).

We note that SLOSS predictions (Table 1) and metacommunity theory (Leibold *et al*., 2004) rely on similar mechanisms. However, to our knowledge, metacommunity theory has not been explicitly used to predict the outcome of SLOSS. In their recent review of the link between metacommunity theory and biodiversity conservation, Chase *et al*. (2020, p. 8) suggest that the “debate in the literature about the influence of habitat fragmentation [of which SLOSS is one element] on biodiversity .. is largely misplaced until one can gain a more definitive focus on the mechanisms being influenced and the scales at which those influences occur.” On the other hand, Fournier *et al*.'s (2017) proposed general theory of metacommunity ecology, while not explicitly aimed at the SLOSS question, indirectly supports our SLOSS cube hypothesis. Fournier *et al*. (2017) modelled the independent and combined effects on species coexistence (here analogous to species richness) of: (*i*) habitat aggregation (analogous to the SS to SL gradient), (*ii*) aggregation of environmental conditions (analogous to across‐habitat heterogeneity), and (*iii*) connectivity (analogous to the frequency of between‐patch movements). They predict maximum species coexistence under the combined conditions of low habitat aggregation (SS), high across‐habitat heterogeneity, and high between‐patch movement frequency. It therefore appears that the SLOSS cube hypothesis is compatible with general metacommunity theory, although a more formal evaluation is needed.

We recognize that even if there is no evidence for SL > SS as a general principle, this will not necessarily translate into a higher priority for preservation of SS than SL *in a given situation*. As mentioned above, factors other than total species richness enter into decisions about habitat preservation and restoration. For example, maintenance of some ecological processes may require large areas, and the perception of wildness may depend on the size of the ecosystem (Perino *et al*., 2019). In addition, a few large areas may be easier to manage than many small ones, as suggested by Higgs & Usher (1980). Finally, when the goal is to conserve a particular threatened species, preserving a large, contiguous area may often be more effective than preserving several small ones. For example, for species that are prone to be killed or removed legally or illegally when encountered or discovered by people (e.g. top predators or high‐value trees), large contiguous natural areas may be the only way to ensure that such encounters are rare (e.g. Müller *et al*., 2014). Nevertheless, in such situations, prioritizing preservation of few large areas over many small ones would be taken not because the SL > SS principle is valid, but in spite of the fact that it is not.

We also note that, if there are no consistent, empirically demonstrated conditions leading to SL > SS, this does not invalidate the mechanisms proposed in Table 1. Rather, it would mean that the mechanisms predicting SS > SL outweigh the mechanisms predicting SL > SS in nature. Put differently, it would mean that, for a mechanism to result in SL > SS, the conditions would need to be so extreme – e.g. perfectly homogeneous habitat and essentially no between‐patch movement – that they are almost never observed except in models.

We emphasize that SLOSS is explicitly not about the role of habitat amount. Rather, it is about the influence of the pattern or configuration of a given amount of habitat (several small *versus* few large patches). Habitat loss is the main cause of species declines, and so habitat preservation and restoration are the top priorities for biodiversity conservation. If, in a particular situation, a choice is presented between conserving one large patch *versus* several small patches, but the small patches have much less habitat in total, then the decision should be to conserve the large patch. For example, in the Steigerwald forest in Southern Germany there is an ongoing discussion about establishing a national park of 10000 ha *versus* protecting a set of smaller areas of about 5000 ha in total. Here, the large national park would probably be more effective for biodiversity conservation, because the total habitat preserved would be twice that of the set of small patches.

We also emphasize that we would never recommend the intentional fragmentation of what is now continuous habitat. Such areas are increasingly rare globally (Watson *et al*., 2016), and their fragmentation would entail loss of habitat. Large protected areas should remain, and to the extent possible, so should remaining large unprotected tracts of contiguous habitat.

## V. CONCLUSIONS

1. While most empirical SLOSS studies find SS > SL, many conservation agencies prioritize protection of large, contiguous areas of habitat, while small patches of natural habitat are less likely to be protected.
2. We suggest that this dilemma can be resolved by asking, ‘are there consistent, empirically demonstrated conditions leading to SL > SS?’
3. Most predictions of SL > SS depend on the assumption that variation in extinction rate dominates the outcome of the extinction–colonization dynamic. This should occur when populations in separate patches are largely independent of each other due to low between‐patch movements, and when species differ in minimum patch size requirements, leading to a strong pattern of species nestedness with patch size.
4. However, even when variation in extinction rate dominates the outcome of the extinction–colonization dynamic, theory can predict SS > SL if extinctions are caused by antagonistic species or disturbances, leading to spreading‐of‐risk of landscape‐scale extinction across SS.
5. SS > SL is also predicted when variation in colonization dominates the outcome of the extinction–colonization dynamic, due to higher immigration rates for SS than SL, and larger species pools in the proximity of SS than SL.
6. Considerations of beta diversity also lead to predictions of SS > SL because SS will intersect or ‘sample’ more micro‐habitats and more species distributions when micro‐habitats and species are clumped or spatially autocorrelated.
7. We summarize these predictions into the SLOSS cube hypothesis, where the combination of three axes – between‐patch movement, the role of spreading‐of‐risk for landscape‐scale population persistence, and across‐habitat heterogeneity – predicts the SLOSS outcome.
8. We use the SLOSS cube hypothesis, combined with existing SLOSS empirical evidence, to predict SL > SS only when all of the following are true: between‐patch movement is low, spreading‐of‐risk is relatively unimportant for landscape‐scale population persistence, and large‐scale across‐habitat heterogeneity is low.
9. Testing this prediction will require a large number of studies targeted at species groups and regions where these three conditions hold.
10. These studies should be designed such that samples are randomly distributed across habitat over multiple equal‐sized landscapes containing different numbers and sizes of patches but the same total amount of habitat.
11. If the majority of studies in these conditions show more species in landscapes with few large than several small patches then this will delineate the situations in which the SL > SS principle can be included as a criterion in reserve design.
12. On the other hand, if the majority of studies in these conditions find more species in landscapes with several small patches, or no difference, then the SL > SS principle should be abandoned.

## Supporting information


**Appendix S1**. Use of the term ‘SLOSS debate’ as a proportion of all English‐language literature, per year since 1980, estimated using *Google Ngram* (Michel *et al*., 2011).
**Appendix S2**. Habitat extent (maximum distance between habitat edges in a landscape) *versus* number of patches in the landscape, for landscapes within each of 32 studies included in the main analysis in Watling *et al*. (2020).
