# Supplementary material for: Resolving the SLOSS dilemma for biodiversity conservation: a research agenda
Source: Biol Rev Camb Philos Soc. 2021 Aug 28;97(1):99–114. doi: 10.1111/brv.12792 (PMC9290967; doi:10.1111/brv.12792)
Supplement: Supplementary file 1 — Appendix S1. Use of the term ‘SLOSS debate’ as a proportion of all English‐language literature, per year since 1980, estimated using Google Ngram (Michel et al., 2011). Appendix S2. Habitat extent (maximum distance between habitat edges in a landscape) versus number of patches in the landscape, for landscapes within each of 32 studies included in the main analysis in Watling et al. (2020). [file BRV-97-99-s001.docx]

**Appendix S1.** Use of the term ‘SLOSS debate’ as a proportion of all English-language literature, per year since 1980, estimated using *Google Ngram* (Michel *et al*., 2011).

**Appendix S2.** Habitat extent (maximum distance between habitat edges in a landscape) *versus* number of patches in the landscape, for landscapes within each of 32 studies included in the main analysis in Watling *et al.* (2020). For each study, each point represents a circular landscape within which habitat extent and patch number were calculated. Landscape size was constant within each study, but varied among studies from about 13 ha to over 11,000 ha. Boxplots in insets are the medians of the total habitat amount per landscape, for landscapes with few (SL, 1–3) *versus* many (SS, > 3) patches; studies without insets had no landscapes with 1–3 patches (SL). The value on the bottom left of the boxplots is the *P* value for the independent samples *t*-test comparing the mean habitat amount (as a per cent of the landscape) in SL *versus* SS landscapes. The strength and direction of the correlation between habitat extent and the number of patches per landscape were inconsistent across studies. This was true for all studies, and for those in which there was no significant difference in habitat area between SL and SS (Studies 3, 14, 19, 26, 28, and 34), i.e. studies appropriate for a SLOSS comparison. Note that three of an original 35 studies were omitted from Watling *et al*. (2020) due to insufficient information, or insufficient variation in predictor variables.
